# Phosphodiesterase 4 inhibitors and db-cAMP inhibit TNF-α release from human mononuclear cells. Effects of cAMP and cGMP-dependent protein kinase inhibitors

**DOI:** 10.1155/S0962935196000580

**Published:** 1996-12

**Authors:** A. Hichami, E. Boichot, N. Germain, E. Berdyshev, O. Coqueret, V. Lagente

**Affiliations:** 1 Laboratoire de Pharmacodynamie et de Pharmacologie Méolculaire Faculté des Sciences Pharmaceutiques et Biologiques Université de Rennes 1 Rennes cedex 35043 France; 2 Institute of Marine Biology Vladivostok Russia; 3 Molecular Oncology Group Royal Victoria Hospital McGill University Montreal, Quebec Canada

## Abstract

We investigated the effects of specific inhibitors of cAMP-dependent protein kinase (PKA) and cGMP-dependent protein kinase (PKG) on the inhibitory activity of phosphodiesterase (PDE) type 4 inhibitors and of the cell permeable analogue of cAMP, db-cAMP on LPS-induced TNF-α release from human mononuclear cells. Incubation from 30 min of mononuclear cells with dbcAMP (10^−5^ to 10^−3^ M), rolipram (10^−9^ M to 10^−5^ M) or Ro 20-1724 (10^−9^ M to 10^−5^ M) significantly inhibited LPS-induced TNF-α release. When mononuclear cells were preincubated for 30 min with the selective PKA inhibitor, H89 (10^−4^ M), but not with the selective PKG inhibitor, Rp-8-pCPT-cGMPs (10^−4^ M), a significant reduction of the inhibitory effect of db-cAMP was noted. Thirty min incubation of mononuclear cells with Rp-8-pCPT-cGMPs induced a significant reduction of the inhibitory activities of both rolipram and Ro 20-1724 (10^−9^ to 10^−5^ M) on LPS-induced TNF-α release, whereas H89 elicited a moderate, but significant inhibition. The present data indicate that db-cAMP inhibits TNF-α release from human mononuclear cells through a PKA-dependent mechanism. In contrast, PDE 4 inhibitors elicit their *in vitro* anti-inflammatory activities *via* a PKG-dependent rather than PKA-dependent activation.


Mediators of Inflammation, 5, 425–428 (1996)

### Phosphodiesterase 4 inhibitors and db-cAMP inhibit TNF-α release from human mononuclear cells. Effects of cAMP and cGMP-dependent protein kinase inhibitors

A. Hichami, E. Boichot, N. Germain, E. Berdyshev,* O. Coqueret** and V. Lagente[CA]

Laboratoire de Pharmacodynamie et de Pharmacologie Moléculaire, Faculté des Sciences Pharmaceutiques et Biologiques, Université de Rennes 1, 35043 Rennes cedex, France; Present Address: *Institute of Marine Biology, Vladivostok, Russia; **Molecular Oncology Group, Royal Victoria Hospital, McGill University, Montreal, Quebec, Canada

[CA]Corresponding Author
Tel: (+33) 99 33 68 50
Fax: (+33) 99 33 62 42/99 33 68 88

**We investigated the effects of specific inhibitors of cAMP-dependent protein kinase (PKA) and cGMP-dependent protein kinase (PKG) on the inhibitory activity of phosphodiesterase (PDE) type 4 inhibitors and of the cell permeable analogue of cAMP, db-cAMP on LPS-induced TNF-α release from human mononuclear cells. Incubation from 30 min of mononuclear cells with db-cAMP ($10^{-5}$ to $10^{-3}$ M), rolipram ($10^{-9}$ M to $10^{-5}$ M) or Ro 20-1724 ($10^{-9}$ M to $10^{-5}$ M) significantly inhibited LPS-induced TNF-α release. When mononuclear cells were preincubated for 30 min with the selective PKA inhibitor, H89 ($10^{-4}$ M), but not with the selective PKG inhibitor, Rp-8-pCPT-cGMPs ($10^{-4}$ M), a significant reduction of the inhibitory effect of db-cAMP was noted. Thirty min incubation of mononuclear cells with Rp-8-pCPT-cGMPs induced a significant reduction of the inhibitory activities of both rolipram and Ro 20-1724 ($10^{-9}$ to $10^{-5}$ M) on LPS-induced TNF-α release, whereas H89 elicited a moderate, but significant inhibition. The present data indicate that db-cAMP inhibits TNF-α release from human mononuclear cells through a PKA-dependent mechanism. In contrast, PDE 4 inhibitors elicit their *in vitro* anti-inflammatory activities *via* a PKG-dependent rather than PKA-dependent activation.**

**Key words:** Cyclic AMP, cyclic GMP, Lipopolysaccharide, Mononuclear cells, Phosphodiesterase, Protein kinase A, Protein kinase G, TNF-α

### Introduction

The nucleotides cyclic 3′,5′adenosine monophosphate (cAMP) and cyclic 3′,5′guanosine monophosphate (cGMP) are important second messengers. Indeed, increased intracellular levels of cAMP or cGMP in both respiratory smooth muscle and inflammatory cells result in bronchodilatation, reduction of inflammation and immunomodulatory activities.[1,2] The intracellular concentration of cyclic nucleotides is mainly determined by intracellular breakdown of cyclic nucleotide by phosphodiesterases (PDEs). PDEs are a family of enzymes which hydrolyse the 3′-ribose phosphate bond of the naturally occurring second messenger nucleotide 3′,5′-cyclic monophosphate to form the biologically inert 5′-nucleotide monophosphate. PDEs are at present divided into at least seven families each with distinct substrate specificities and regulatory characteristics.[3,4] PDE 3 and PDE 4 are specifically responsible for cAMP hydrolysis, whilst PDE 5 is cGMP specific.

The physiological actions of cAMP and cGMP are claimed to be mediated by cAMP-dependent protein kinase (PKA) and cGMP-dependent protein kinase (PKG), respectively.[5] This phenomenon has been observed in many tissues, where the activation state of PKA or PKG is usually associated with the elevation of its corresponding cyclic nucleotide, cAMP or cGMP.[5,6] However, partly because of a lack of absolute specificity of either enzyme and of the relatively high level of cAMP or cGMP in certain tissues, it is also possible that either cyclic nucleotide can cross-activate the other kinase.[7]

It has been previously demonstrated that the PDE 4 inhibitors, rolipram and Ro 20-1724, markedly reduced mononuclear cell activation such as inhibition of fMLP-induced arachidonate release and LPS-induced TNF-α release from human peripheral blood mononuclear cells.[8–11] Whether or not increase in intracellular cAMP is involved in the anti-inflammatory activities of PDE 4 inhibitors may be further clarified. In order to evaluate the respective contribution of the activation of protein kinases, we analysed the effects of specific inhibitors of PKA and

© 1996 Rapid Science Publishers

PKG on the inhibitory activities of PDE 4 inhibitors, rolipram and Ro 20-1724 and the cell permeable analogue of cyclic AMP, db-cAMP on TNF-α release from human monocytes.

### Methods

### Materials

The following agents and drugs were used: Ficoll-Hypaque (Pharmacia, Upsala, Sweden), phosphate buffered saline without $Ca^{2+}/Mg^{2+}$ (PBS), RPMI 1640, glutamine, penicillin and streptomycin (Gibco, Cergy-Pontoise, France), fetal calf serum (Flow Laboratories, Irvine, UK), bovine serum albumin (BSA), lipopolysaccharide (LPS) from *Escherichia coli* 055:B5, dimethylsulphoxide (DMSO), dibutyryl cyclic AMP (db-cAMP) (Sigma, St Louis, MO, USA), Ro 20-1724 (RBI, Natick, MA, USA), H89 (Calbiochem, San Diego, USA), Rp-8-pCPT-cGMPs (BIOLOG Life Science Institute, Bremen, Germany). Rolipram was synthetized at the Institut de Recherche Jouveinal (Fresnes, France).

### Preparation of human mononuclear cells

Mononuclear cells were isolated from fresh buffy coats obtained from healthy donors by density gradient centrifugation on Ficoll-Hypaque as previously described.[8] Cells ($1.5 \times 10^6$ cells/ml) were seeded in 24-well Petri dishes and cultured for 12 h (FALCON, Franklin Lakes, NJ, USA) in RPMI-FCS (10%) at 37°C in a 5% $CO_2$ and 95% humidity atmosphere with or without the indicated stimuli.

### TNF-α production

After stimulation with LPS (10 μg/ml), cell-free supernatants were collected, centrifugated (2000 *g*) and stored frozen at −20°C prior to TNF-α determination. TNF-α concentrations in cell culture supernatants were determined by specific ELISA using a commercial kit (Genzyme Corp., Cambridge, MA, USA). Sensitivity of the assay was 1 pg/ml. The absorbance at 450 nm was assessed with an ELISA reader (Dynatech, Alexandria, VA, USA).

### Drug treatment

Mononuclear cells were incubated for 30 min with H89 ($10^{-4}$ M) or Rp-8-pCPT-cGMPs ($10^{-4}$ M), then they were treated for 30 min with db-cAMP ($10^{-5}$ M to $10^{-3}$ M) or with one of the selective PDE 4 inhibitors, rolipram ($10^{-9}$ to $10^{-5}$ M) or Ro 20-1724 ($10^{-9}$ to $10^{-5}$ M). All drugs, were dissolved in RPMI supplemented with 0.2% free fatty acid BSA with the exception of PDE inhibitors which were dissolved in dimethylsulphoxide (DMSO, Sigma, 0.1%, final concentration). Vehicle controls were included in the experimental design.

### Data analysis

Results are expressed as percentage of control of TNF-α ± S.E.M. of four to five experiments, done in triplicate. Analysis for statistical significance was done by paired Student *t*-test.

### Results

Incubation of mononuclear cells with db-cAMP ($10^{-5}$ to $10^{-3}$ M), rolipram ($10^{-9}$ M to $10^{-5}$ M) or Ro 20-1724 ($10^{-9}$ M to $10^{-5}$ M) for 30 min concentration-dependently inhibited LPS-induced TNF-α release (Figs 1–3). Fig. 1 also shows that in the presence of the selective PKA inhibitor, H89 ($10^{-4}$ M) for 30 min, a significant reduction of the inhibitory effect of db-cAMP ($10^{-4}$ M to $10^{-3}$ M) was noted. In contrast, preincubation of the cells with the selective PKG inhibitor, Rp-8-cpt-cGMPs ($10^{-4}$ M), failed to significantly modify the inhibitory activity of db-cAMP on LPS-induced TNF-α release (Fig. 1).

When mononuclear cells were preincubated for 30 min with H89 ($10^{-4}$ M), a moderate inhibition of the effect of PDE 4 inhibitors, rolipram and Ro 20-1724 was observed and

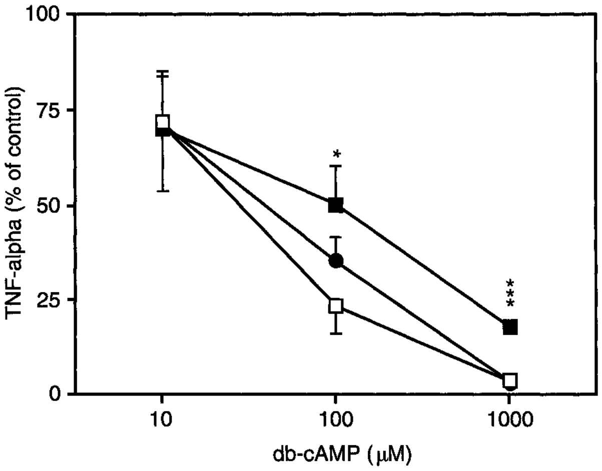

FIG. 1. Effects of H89 (■, $10^{-4}$ M) or Rp-8-pCPT-cGMPs (●, $10^{-4}$ M) on the inhibitory activity of db-cAMP ($10^{-5}$ to $10^{-3}$ M) on LPS-induced TNF-α release from human mononuclear cells. Results are expressed as percentage of control of TNF-α ± S.E.M. of four to five experiments done in triplicate. *$p < 0.05$, ***$p < 0.001$ compared with the responses of mononuclear cells incubated with db-cAMP alone (control: □).

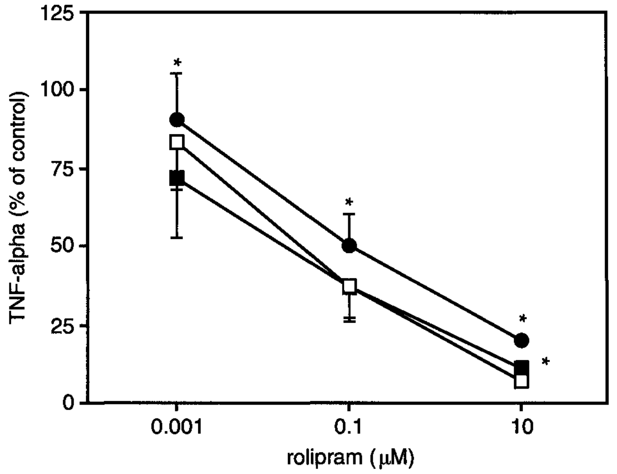

FIG. 2. Effects of H89 (■, $10^{-4}$ M) or Rp-8-pCPT-cGMPs (●, $10^{-4}$ M) on the inhibitory activity of rolipram ($10^{-9}$ to $10^{-5}$ M) on LPS-induced TNF-α release from human mononuclear cells. Results are as percentage of control of TNF-α ± S.E.M. of four to five experiments done in triplicate. *$p < 0.05$, compared with the responses of mononuclear cells incubated with rolipram alone (control: □).

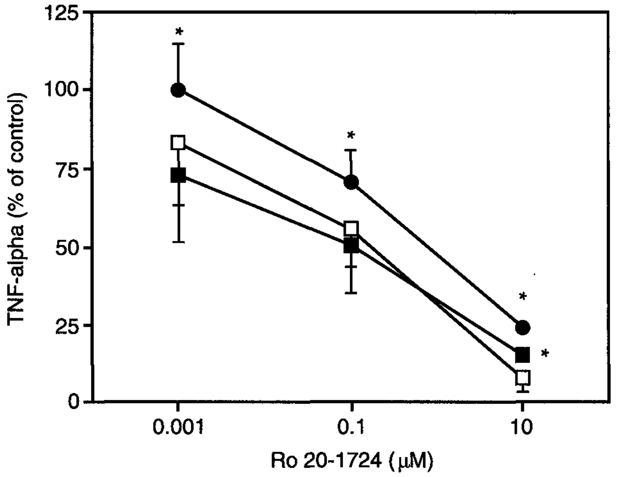

FIG. 3. Effects of H89 (■, $10^{-4}$ M) or Rp-8-pCPT-cGMPs (●, $10^{-4}$ M) on the inhibitory activity of Ro 20-1724 ($10^{-9}$ to $10^{-5}$ M) on LPS-induced TNF-α release from human mononuclear cells. Results are as percentage of control of TNF-α ± S.E.M. of four to five experiments done in triplicate. *$p < 0.05$, compared with the responses of mononuclear cells incubated with Ro 20-1724 alone (control: □).

appeared significant for $10^{-5}$ M rolipram and Ro 20-1724 ($p < 0.05$) (Figs 2 and 3). Hence, 30 min incubation of mononuclear cells with Rp-8-cpt-cGMPs ($10^{-4}$ M) induced a significant reduction of the inhibitory activities of both rolipram and Ro 20-1724 ($10^{-9}$ M to $10^{-5}$ M) on LPS-induced TNF-α release (Figs 2 and 3).

### Discussion

It has long been known that cAMP is an important second messenger in a wide variety of cell systems. In general, intracellular elevation of cAMP level is thought to inhibit the function of inflammatory cells. Hence, adenylyl cyclase stimulating agents or PDE inhibitors markedly reduced LPS-induced TNF-α production from mononuclear cells in various experimental conditions.[9–13] Nevertheless, PDE inhibitors induced only a small increase of cAMP despite pronounced TNF-α suppression in LPS-stimulated PBMC.[14] We previously demonstrated that PDE 4 inhibitors dose-dependently reduced the fMLP-induced arachidonate release from mononuclear cells.[8] However, this effect is not closely associated with a rise in intracellular cAMP, even though a significant enhancement of intracellular cAMP has been observed after incubation of the cells with PDE 4 inhibitors plus forskolin, an activator of adenylyl cyclase.[8] Compartmentalization is a possible reason for low overall levels of cAMP with PDE inhibitors. Moreover, we recently observed that the selective PKA inhibitor, H89 did not significantly reduce the inhibitory activity of db-cAMP and PDE 4 inhibitors of fMLP-induced arachidonate release from human monocytes.[15] Therefore, we investigated the possible interactions of PKA and PKG inhibitors on the activity of PDE 4 inhibitors on LPS-induced TNF-α release from mononuclear cells.

The present research confirmed that the cell permeable analogue of cAMP, db-cAMP and the PDE 4 inhibitors, rolipram and Ro 20-1724 concentration-dependently inhibited the LPS-induced TNF-α release from mononuclear cells. As expected, the present data also showed that the selective PKA inhibitor H89[16,17] reduced the inhibition of TNF-α release from human monocytes elicited by db-cAMP, whereas the selective PKG inhibitor Rp-8-pCPT-cGMPs[18] was not markedly effective. These results suggest that PKA activation is mainly involved in the mechanism of action of db-cAMP in mononuclear cells.

Rp-8-pCPT-cGMPs significantly reduced the inhibitory activities of the two PDE 4 inhibitors, rolipram and Ro 20-1724, whereas H89 only elicited moderate activities which was only statistically significant at the highest concentration of PDE 4 inhibitors. Therefore, the present data demonstrated a mechanism insensitive to H89 and therefore independent of PKA for rolipram and Ro 20-1724. Similar observations have been recently reported, where cyclic AMP-elevating agents and db-cAMP prolonged eosinophil survival by mechanisms insensitive to H89 and therefore PKA-independent.[19] In contrast, the results obtained with Rp-8-pCPT-cGMPs suggest an involvement of PKG in the mechanism of action of PDE 4 inhibitors.

Until recently, it was accepted that cAMP and cGMP activate their respective kinases with a high degree of specificity. The validity of this assumption has steadily declined over the past decade and it now appears that there is evidence for cross-activation of PKA and PKG by adenyl and guanyl 3′:5′-cyclic nucleotides (for review see Ref. 20). Moreover, their cyclic nucleotide binding domains share a high degree of amino acid sequence identity, since only a single alanine–threonine difference between their cAMP- and cGMP-binding domains partially accounts for this specificity.[7,21] Such a cross-activation occurred in airway smooth muscle cells where cAMP activates PKG with an $EC_{50}$ of 80 nM, which is slightly different than the cAMP $EC_{50}$ for PKA (30 nM).[22] Involvement of PKG in cAMP mediated actions has been also reported for the cardiovascular system. For example, it has been reported that the increase in cAMP content following treatment of bovine coronary arteries with isoproterenol results in a simultaneous activation of both PKA and PKG.[23] Moreover, an important role for PKG in mediating the relaxant response to cAMP have been noted in rat aortic smooth muscle cells in culture.[24] Recently, Eckly and colleagues[25] reported that PKG is involved in rolipram-induced vascular relaxation of rat aorta. Since rolipram only increased cAMP content, these data also suggest a cross-activation of PKG by cAMP in rat aorta.

In conclusion, we showed that db-cAMP inhibits TNF-α release from human mononuclear cells through a PKA-dependent mechanism. Furthermore, the selective PKG inhibitor, Rp-8-pCPT-cGMPs but not the selective PKA inhibitor, H89 significantly reduced the inhibitory activity of PDE 4 inhibitors on LPS-induced TNF-α release from human mononuclear cells. Therefore, these results suggest a PKA-independent and a PKG-dependent mechanism for the *in vitro* anti-inflammatory activity of PDE 4 inhibitors, which may be further clarified.

### References

1. Nicholson CD, Shahid M. Inhibitors of cyclic nucleotide phosphodiesterase isoenzymes—their potential utility in the therapy of asthma. *Pulm Pharmacol* 1994; **7**: 1–17.
2. Coqueret O, Demarquay D, Lagente V. Role of cyclic AMP in the modulation of IgE production by the $\beta_2$-adrenoceptor agonist, fenoterol. *Eur Respir J* 1996; **9**: 220–225.
3. Beavo JA, Reifsnyder DK. Primary sequence of cyclic nucleotide phosphodiesterase isoenzymes and the design of selective inhibitors. *Trends Pharmacol Sci* 1990; **11**: 150–155.
4. Beavo JA, Conti M, Heaslip RJ. Multiple cyclic nucleotide phosphodiesterase. *Mol Pharmacol* 1994; **46**: 399–405.
5. Lincoln TM, Corbin JD. Characterization and biological role of the cGMP-dependent protein kinase. *Adv Cyclic Nucleotide Res* 1983; **15**: 139–192.
6. Beavo JA, Bechtel PJ, Krebs EG. Activation of protein kinase by physiological concentrations of cyclic AMP: kinetics at high enzyme concentrations. *Proc Natl Acad Sci USA* 1974; **71**: 3580–3583.
7. Jiang H, Shabb JB, Corbin JD. Cross-activation: overriding cAMP/cGMP selectivities of protein kinases in tissues. *Biochem Cell Biol* 1992; **70**: 1283–1289.
8. Hichami A, Boichot E, Germain N, Legrand A, Moodley I, Lagente V. Involvement of cyclic AMP in the effects of phosphodiesterase IV inhibitors on arachidonate release from mononuclear cells. *Eur J Pharmacol Mol Pharmacol section* 1995; **291**: 91–97.
9. Prabhakar U, Lipshutz D, O'Leary Bartus J, Slivjak MJ, Smith EF, Lee JC, Esser KM. Characterization of cyclic AMP-dependent inhibition of LPS-induced TNF-α production by rolipram, a specific phosphodiesterase IV (PDE IV) inhibitor. *Int J Immunopharm* 1994; **16**: 805–816.
10. Molnar-Kimber KL, Yonno L, Heaslip RJ, Weichman BM. Differential regulation of TNF-α and IL-1β production from endotoxin stimulated human monocytes by phosphodiesterase inhibitors. *Med Inflamm* 1992; **1**: 411–417.
11. Semmler J, Wachtel H, Enders S. The specific type IV phosphodiesterase inhibitor rolipram supresses tumor necrosis factor-α production by human mononuclear cells. *Int J Immunopharmacol* 1993; **15**: 409–413.
12. Verghese MW, McConnell RT, Strickland AB, Gooding RC, Stimpson SA, Yarnall DP, Taylor JD, Furdon PJ. Differential regulation of human monocyte-derived TNF-α and IL-1β by type IV cAMP-phosphodiesterase (cAMP-PDE) inhibitors. *J Pharmacol Exp Therap* 1995; **272**: 1313–1320.
13. Seldon PM, Barnes PJ, Meja K, Giembycz MA. Suppression of lipopolysaccharide-induced tumor necrosis factor-α generation from human peripheral blood monocytes by inhibitors of phosphodiesterase 4: interaction with stimulants of adenylyl cyclase. *Mol Pharmacol* 1995; **48**: 747–757.
14. Sinha B, Semmler J, Eisenhut T, Eigler A, Endres S. Enhanced tumor necrosis factor supression and cyclic adenosine monophosphate accumulation by combination of phosphodiesterase inhibitors and prostanoids. *Eur J Immunol* 1995; **25**: 147–153.
15. Hichami A, Boichot E, Germain N, Coqueret O, Lagente V. Interactions between cAMP- and cGMP-dependent protein kinase inhibitors and phosphodiesterase 4 inhibitors on arachidonate release from human monocytes. *Life Sci* 1996; **59**: PL255–PL261.
16. Hidaka H, Inagaki M, Kawamoto S, Sasaki Y. Isoquinolinesulfonamides, novel and potent inhibitors of cyclic nucleotide dependent protein kinase and protein kinase C. *Biochemistry* 1984; **23**: 5036–5041.
17. Chijiwa T, Mishima A, Hagiwara M, Sano M, Hayashi K, Inoue T, Naito K, Toshioka T, Hidaka H. Inhibition of forskolin-induced neurite outgrowth and protein phosphorylation by a newly synthetized selective inhibitor of cyclic-AMP-dependent protein kinase, N-[2-(p-bromocinnamylamino)ethyl]-5-isoquinolinesulfonamide (H89), of PC12D pheochromocytoma cells. *J Biol Chem* 1990; **265**: 5267–5272.
18. Butt E, Eigenthaler M, Genieser HG. (RP)-8-pCPT-cGMPs. A novel cGMP-dependent protein kinase inhibitor. *Eur J Pharmacol – Mol Pharmacol section* 1994; **269**: 265–268.
19. Hallsworth MP, Giembycz MA, Barnes PJ, Lee TH. Cyclic AMP-elevating agents prolong or inhibit eosinophil survival depending on prior exposure to GM-CSF. *Br J Pharmacol* 1996; **117**: 79–86.
20. Schmidt HH, Lohmann SM, Walter U. The nitric oxide and cGMP signal transduction system: regulation and mechanism of action. *Biochem Biophys Acta* 1993; **1178**: 153–175.
21. Francis SH, Corbin JD. Structure and function of cyclic nucleotide-dependent protein kinases. *Annu Rev Physiol* 1994; **56**: 237–272.
22. Torphy TJ, Freese W, Rinard GA, Brunton LL, Mayer SE. Cyclic nucleotide-dependent protein kinases in airway smooth muscle. *J Biol Chem* 1982; **257**: 11609–11616.
23. Jiang H, Colbran JL, Francis SH, Corbin JD. Direct evidence for cross-activation of cGMP-dependent protein kinase by cAMP in pig coronary arteries. *J Biol Chem* 1992; **267**: 1015–1019.
24. Lincoln TM, Cornwell TL, Taylor AE. cGMP-dependent protein kinase mediates the reduction of $Ca^{2+}$ by cAMP in vascular smooth muscle cells. *Am J Physiol* 1990; **258**: C399–C407.
25. Eckly AE, Stoclet JC, Lugnier C. Involvement of protein kinase G in vascular relaxation induced by the cyclic nucleotide phosphodiesterase IV inhibitor rolipram. *Br J Pharmacol* 1995; **114**: 206P.

ACKNOWLEDGEMENTS. The authors thank Dr Tardivel and Mrs Massot (CRTS Rennes) for the supply of buffy coats.

**Received 10 July 1996; accepted in revised form 27 September 1996**
